# Prognostic value of an automated bone scan index for men with metastatic castration-resistant prostate cancer treated with cabazitaxel

**DOI:** 10.1186/s12885-018-4401-y

**Published:** 2018-05-02

**Authors:** Koichi Uemura, Yasuhide Miyoshi, Takashi Kawahara, Jikuya Ryosuke, Daisuke Yamashita, Shuko Yoneyama, Yumiko Yokomizo, Kazuki Kobayashi, Takeshi Kishida, Masahiro Yao, Hiroji Uemura

**Affiliations:** 1 Department of Urology and Renal Transplantation, Yokohama City University Medical Center, 4-57 Urafune-cho, Minami-ku, Yokohama, 2320024 Japan; 2 Department of Urology, Yokohama City University Graduate School of Medicine, Yokohama, Japan; 3 Department of Urology, Yokosuka Kyosai Hospital, Yokosuka, Japan; 4 Department of Urology, Kanagawa Cancer Center, Yokohama, Japan; 5 Department of Urology, Yokohama City Minato Red Cross Hospital, Yokohama, Japan

**Keywords:** Prostate cancer, Castration-resistant, Survival prediction, Bone scan index

## Abstract

**Background:**

A computer-assisted diagnostic system for analyzing bone scans (BONENAVI) calculates the automated bone scan index (aBSI). Here we evaluated the aBSI as a prognostic imaging biomarker for men with metastatic castration-resistant prostate cancer (mCRPC) treated with cabazitaxel.

**Methods:**

We retrospectively analyzed 48 patients who received cabazitaxel for mCRPC and evaluated the ability of the aBSI to predict overall survival (OS). The Cox proportional hazards model was used to investigate the associations between baseline aBSI at cabazitaxel treatment and OS with the clinical variables as follows: age, number of cycles of docetaxel, serum prostate-specific antigen, hemoglobin (Hb), lactate dehydrogenase (LDH), and alkaline phosphatase. We determined the C-index to evaluate the discriminatory ability of our models when we included or excluded the aBSI from the analyses.

**Results:**

The median OS after cabazitaxel treatment was 10.0 months, and patients with aBSI ≤1% achieved significantly longer OS compared with patients with aBSI ≥1%. Multivariate analysis showed that age, Hb, LDH, and aBSI were independent prognostic factors of OS. Adding aBSI to the base model increased the C-index from 0.78 to 0.80.

**Conclusions:**

The aBSI may serve as a useful imaging biomarker for predicting OS among men with mCRPC treated with cabazitaxel. Prospective studies are required to establish the value of aBSI as prognostic imaging biomarker.

## Background

Although 80%–90% of prostate cancers with metastasis respond to initial hormone therapy, most patients finally develop to metastatic castration-resistant prostate cancer (mCRPC) [1, 2]. Sipuleucel-T, abiraterone acetate, enzalutamide, docetaxel, cabazitaxel, and radium-223 have all improved survival among men with mCRPC and have been approved in western countries [3, 4].

Cabazitaxel is the first chemotherapeutic agent to prolong overall survival (OS) of patients with mCRPC who are administered docetaxel. A phase III randomized controlled clinical trial (TROPIC) found that cabazitaxel prolongs OS compared with mitoxantrone and reduces the relative risk of death by 30% [5]. Accordingly, cabazitaxel is widely used for patients with mCRPC. However, no clinical biomarkers are available for predicting survival.

A computer-assisted diagnostic system for analyzing bone scans (BONENAVI; Fujifilm RI Pharma Co. Ltd., Tokyo, Japan) calculates the automated bone scan index (aBSI) that provides an objective and quantitative evaluation of the burden imposed by bone metastasis [6]. The prognostic value of aBSI as a biomarker was evaluated for predicting the survival of men treated with life-prolonging agents such as enzalutamide [7], abiraterone [7, 8], docetaxel [9–11], and radium-223 [12]. Here we conducted a retrospective analysis of the relationship between OS and baseline aBSI of patients with mCRPC treated with cabazitaxel.

## Methods

### Study design, patients, and treatment

We retrospectively analyzed 48 patients who were treated with cabazitaxel for bone-metastatic mCRPC between 2014 and 2016 at Yokohama City University Medical Center, Yokohama City University Hospital, and their associated hospitals. All patients had histologically confirmed prostate adenocarcinoma. The 2009 TNM clinical staging system and the 2005 International Society of Urologic Pathology Gleason grading system were used [13]. The clinical stages of all patients were evaluated using chest and body computed tomography as well as bone scans upon initiation of cabazitaxel treatment.

All patients were initially treated with androgen deprivation therapy (medical or surgical castration with an antiandrogen). After failure to CRPC, all patients were administered enzalutamide, abiraterone, or both, followed by docetaxel with dexamethasone, before cabazitaxel was administered. All patients received continuous androgen ablation therapy (an LH-RH analog) and oral dexamethasone (0.5–1.0 mg). Cabazitaxel treatment was continued until disease progression or unacceptable adverse events occurred.

Some patients received bisphosphonate or denosumab after the development of mCRPC. Patients were not administered sipuleucel-T and radium-223. For terminally ill patients, palliative therapy and pain control using morphine and palliative external beam radiotherapy were used as appropriate. Serum prostate-specific antigen (PSA) levels were measured using the Elecsys Total PSA Assay (Roche Diagnostics Corp., Basle, Switzerland).

### Bone scan index

Bone scan images were obtained within 1 month before or after initiating cabazitaxel treatment and analyzed using BONENAVI version 2 [14]. The automated method for analysis of anterior and posterior whole-body bone scan images was previously described [15]. The aBSI was calculated as the percentage of the sum of all hot-spots classified as bone metastases using the artificial neural network (ANN) values [14].

### Statistical analysis

The Cox proportional hazards model with stepwise regression analysis was used to investigate the association between aBSI, OS, and clinical variables of patients treated with cabazitaxel as follows: age, number of cycles of docetaxel, serum PSA, hemoglobin (Hb), lactate dehydrogenase (LDH), and alkaline phosphatase (ALP). The cut-off values for age, number of cycles of docetaxel, serum PSA, Hb, LDH, and ALP were determined according to the median value of each variable. The cut-off value of aBSI was defined as 1.0% according to published studies [10, 15]. The relative risks and 95% confidence intervals (CIs) were calculated, and the C-index was used to assess the discriminatory ability of our models.

The Kaplan–Meier product-limit was used to estimate the survival distribution. The log-rank test was used for the analysis of survival differences. All statistical tests were two-sided, and the significance level was defined as alpha = 0.05. All analyses were conducted using IBM SPSS Statistics software for Windows, version 24 (IBM Corp., Armonk, NY, USA) and the R package (R Foundation for Statistical Computing, Vienna, Austria).

The experimental procedures were conducted in accordance with the ethical standards of the Helsinki Declaration.

## Results

**Fig. 1:**
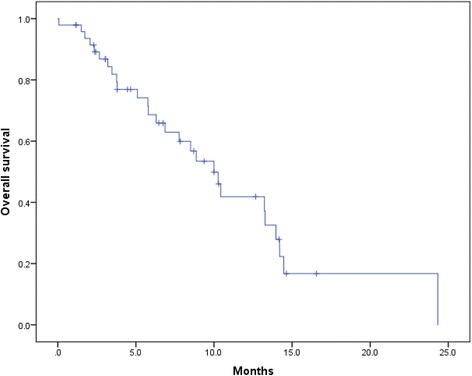
Kaplan–Meier curve of overall survival (OS) after administration of cabazitaxel. The median OS after cabazitaxel was 10.0 months (95% confidence interval [CI], 7.8–12.2 months)

**Fig. 2:**
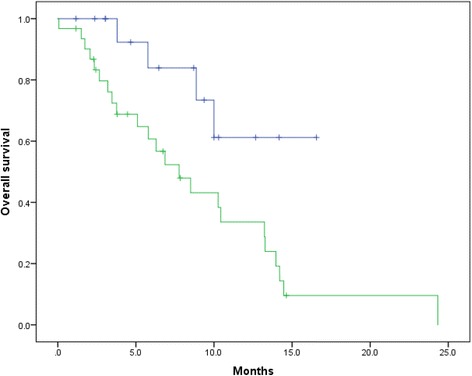
Kaplan–Meier curves of OS after administration of cabazitaxel according to the automated bone scan index (aBSI). The blue line indicates survival of patients with aBSI < 1% (*n* = 17), and the green line indicates survival of patients with aBSI ≥1% (*n* = 31). The median OS of patients with aBSI < 1% and aBSI ≥1% was not reached and 7.8 months (95%CI, 4.6–10.9), respectively (*p* = 0.027)

Patients’ characteristics are shown in Table 1. Among the 48 patients, the sites of metastasis were as follows: 13, only bone; 12, lymph node + bone; 3, visceral + bone; and 20, visceral + lymph node + bone. The median baseline PSA value was 152.1 ng/ml, and the median aBSI was 3.5% (range, 0.0%–12.9%). Twenty-five (56.2%) patients died, and all deaths were caused by prostate cancer. The median OS after cabazitaxel treatment was initiated was 10.0 months (95%CI, 7.8–12.2) (Fig. 1). A Kaplan–Meier curve of OS after cabazitaxel treatment as a function of aBSI is shown in Fig. 2. The median OS of patients with aBSI < 1% (*n* = 17) and aBSI ≥1% (*n* = 31) was not reached and 7.8 months (95%CI: 4.6–10.9), respectively. Patients with aBSI < 1% had a significantly longer OS compared with patients with aBSI ≥1% (*p* = 0.027).

**Table 1:** Patients’ characteristics

Variables	
Median age, years (range)	71.2 (52.5–82.9)
Cycles of docetaxel, cycles (range)	9 (1–55)
Use of enzalutamide, n (%)	35 (72.9)
Use of abiraterone acetate, n (%)	26 (54.1)
Median aBSI, %, (range)	3.5 (0.0–12.9)
Median baseline PSA, ng/mL (range)	152.1 (1.6–3564.0)
Median baseline Hb, g/dL (range)	11.0 (8.1–14.2)
Median baseline LDH, IU/L (range)	262 (124–3509)
Median baseline ALP, IU/L (range)	414 (111–3653)
Lymph node metastasis, n (%)	32 (66.6)
Visceral metastasis, n (%)	23 (47.9)
Cycles of cabazitaxel, cycles (range)	4 (1–15)
Median observation period, months (range)	7.2 (0.6–25.0)
Cancer death, n (%)	25 (56.2)

*aBSI* automated bone scan index, *ALP* alkaline phosphatase, *Hb* hemoglobin, *LDH* lactate dehydrogenase, *PSA* prostate-specific antigen

Univariate analysis revealed that age (hazard ratio [HR], 2.6; 95%CI, 1.1–6.0; *p* = 0.023), Hb (HR, 3.6; 95%CI, 1.6–8.1; *p* = 0.002), LDH (HR, 3.0; 95%CI 1.3–6.5; *p* = 0.008), and aBSI (HR, 3.1; 95%CI, 1.1–9.2; *p* = 0.036) were prognostic factors for OS (Table 2). Multivariate analysis demonstrated that age (HR, 3.4; 95%CI, 1.3–9.1; *p* = 0.013), Hb (HR, 2.6; 95%CI, 1.1–6.2; *p* = 0.034), LDH (HR, 3.3; 95%CI, 1.3–8.6; *p* = 0.014), and aBSI (HR, 5.8; 95%CI, 1.1–30.3; *p* = 0.031) were independent prognostic factors of OS (Table 3).

**Table 2:** Univariate analysis of predictions of overall survival of men with metastatic castration-resistant prostate cancer treated with cabazitaxel

	HR	95% CI		*P* value
		Lower	Upper	
Age (≥71.2 years vs < 71.2)	2.6	1.1	6.0	0.023
Number of cycles of docetaxel (≥10 cycles vs ≤9)	0.8	0.4	1.7	0.533
Baseline aBSI (≥1.0% vs < 1.0)	3.1	1.1	9.2	0.036
Baseline PSA (≥152.1 ng/mL vs < 152.1)	1.7	0.7	3.7	0.218
Baseline Hb (< 11.0 g/dL vs ≥11.0)	3.6	1.6	8.1	0.002
Baseline LDH (≥262 IU/L vs < 262)	3.0	1.3	6.5	0.008
Baseline ALP (≥414 IU/L vs < 414)	2.0	0.9	4.5	0.100

*aBSI* automated bone scan index, *ALP* alkaline phosphatase, *Hb* hemoglobin, *LDH* lactate dehydrogenase, *PSA* prostate-specific antigen

**Table 3:** Multivariate analysis of predictions of overall survival of men with metastatic castration-resistant prostate cancer treated with cabazitaxel

	HR	95% CI		*P* value
		Lower	Upper	
Age (≥71.2 years vs < 71.2)	3.4	1.3	9.1	0.013
Number of cycles of docetaxel (≥10 cycles vs ≤9)	0.8	0.3	2.8	0.781
Baseline aBSI (≥1.0% vs < 1.0)	5.8	1.1	30.3	0.031
Baseline PSA (≥152.1 ng/mL vs < 152.1)	0.8	0.2	2.9	0.769
Baseline Hb (< 11.0 g/dL vs ≥11.0)	2.6	1.1	6.2	0.034
Baseline LDH (≥262 IU/L vs < 262)	3.3	1.3	8.6	0.014
Baseline ALP (≥414 IU/L vs < 414)	0.5	0.2	1.6	0.255

*aBSI* automated bone scan index, *ALP* alkaline phosphatase, *Hb* hemoglobin, *LDH* lactate dehydrogenase, *PSA* prostate-specific antigen

We evaluated the discriminatory ability of our models by determining the C-index. The C-index in our model when aBSI was included was 0.80 for predicting OS after cabazitaxel treatment was initiated. When we analyzed the discriminatory ability of the model after excluding aBSI, we found that the C-index was 0.78 for predicting OS.

**Fig. 3:**
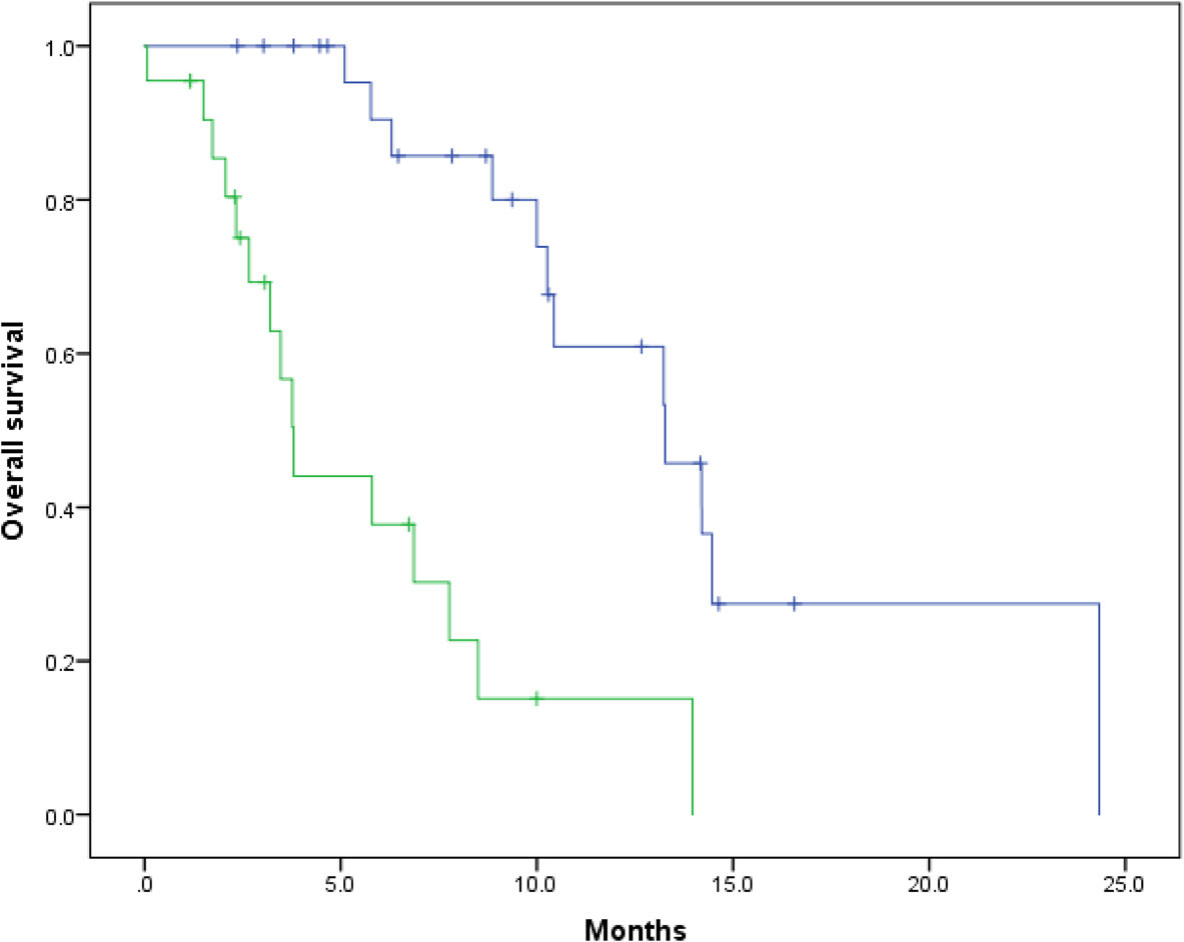
Kaplan–Meier curves of OS after initiating cabazitaxel treatment according to risk group. We stratified the patients into cohorts at low risk (0–2 risk factor, *n* = 26) and high risk (3–4 risk factors, *n* = 22). The blue and green lines indicate survival of patients at low and high risk, respectively. The median OS values of patients at low and high risk were 13.3 months and 3.8 months, respectively (*p* < 0.001). The incidences of the risk factors of each group are shown in Table 4

Finally, we stratified the patients into cohorts at low risk (0–2 risk factor, *n* = 26) or high risk (3–4 risk factors, *n* = 22) (Table 4). There was a statistically significant difference in OS between these groups (*p* < 0.001) (Fig. 3).

**Table 4:** Distributions of the risk factors in the low and high risk groups

	Age ≥ 71.2 years	Hb < 11.0 g/dL	LDH ≥ 262 IU/L	aBSI≥1.0%
Low risk (*n* = 26), n (%)	8 (30.8)	5 (19.2)	6 (23.1)	13 (50.0)
High risk (*n* = 22), n (%)	16(72.7)	18 (81.8)	18 (81.8)	18 (81.8)

*aBSI* automated bone scan index, *Hb* hemoglobin, *LDH* lactate dehydrogenase

## Discussion

The aBSI is useful imaging biomarker for predicting the survival of men with prostate cancer, including those with hormone-naïve prostate cancer or mCRPC [6, 9, 11, 16–20]. For example, the survival of patients with mCRPC with aBSI ≥3% is shorter compared with men with aBSI < 3% who are treated with taxane-based chemotherapy [9]. Further, men with mCRPC with aBSI > 1% survive for shorter times compared with those with aBSI ≤1% who are treated with docetaxel [10]. Moreover, the aBSI is superior compared with the EOD score as a prognostic imaging biomarker [10].

Recent study demonstrated that not only baseline aBSI before treatment, but also aBSI change after treatment could be useful for prognostic imaging biomarker. Miyoshi et al. reported that decreased aBSI after abirateone acetate or enzalutamide was an independent predictor for longer OS among men with mCRPC [7]. Reza et al. also reported that aBSI change after abirateone acetate was related to survival time in mCRPC patients [8]. On the other hand, Fosbol et al. reported that aBSI was a prognostic biomarker for mCRPC patients received with Ra-223, although there was no significant association between aBSI change during Ra-223 therapy and OS [12].

Here we analyzed the association between OS, aBSI, and clinical variables of patients with mCRPC who were treated with cabazitaxel. We identified age, Hb, LDH, and aBSI as independent prognostic factors of OS. Including the aBSI in the base model improved the C-index from 0.78 to 0.80. Therefore, the aBSI may serve as a promising imaging biomarker for predicting OS among men with mCRPC who are treated with cabazitaxel. Further, the aBSI may provide useful information about such patients.

Although we show here that the aBSI played an important role in evaluating bone metastases, our study has several limitations. For example, our study was retrospective, the number of subjects was small, and the observation periods were relatively short. Prospective evaluation of larger patient populations and longer observations are warranted to establish the usefulness of the aBSI as a prognostic imaging biomarker.

## Conclusion

Our analyses strongly indicate that the aBSI may serve as a useful tool for risk stratification of patients with mCRPC undergoing treatment with cabazitaxel.

## Abbreviations

aBSI: Automated bone scan index
ALP: Alkaline phosphatase
CI: Confidence interval
EOD: Extent of disease
Hb: Hemoglobin
HR: Hazard ratio
LDH: Lactate dehydrogenase
mCRPC: Metastatic castration-resistant prostate cancer
OS: Overall survival
PSA: Prostate-specific antigen
